# The value of cytoreductive nephrectomy on the survival of metastatic renal carcinoma patients based on the number of site-specific metastases

**DOI:** 10.1371/journal.pone.0215861

**Published:** 2019-04-23

**Authors:** Zhijian Zhao, Wenqi Wu, Xiaolu Duan, Guohua Zeng, Yongda Liu

**Affiliations:** 1 Department of Urology, Minimally Invasive Surgery Center, The First Affiliated Hospital of Guangzhou Medical University, Guangzhou, China; 2 Guangzhou Institute of Urology, Guangzhou, China; 3 Guangdong Key Laboratory of Urology, Guangzhou, China; University of Wisconsin, UNITED STATES

## Abstract

**Background:**

There has been significant uncertainty in the selection of candidates for cytoreductive nephrectomy (CN) in patients with metastatic renal cell carcinoma (mRCC). This report investigates the influence of site-specific metastases (bone, brain, liver, and lung) on the survival benefit of CN.

**Methods:**

Within the Surveillance, Epidemiology and End Results database (2010–2014), 1113 mRCC patients treated with CN (n = 618) or no surgery (NS, n = 495) met the selection criteria. 168 pairs of patients using propensity scores were matched to balance the selection bias of undergoing CN. Multivariable competing risks regression analysis was used to calculate cancer-specific mortality (CSM) and overall survival (OS). Cases were subdivided to investigate the advantages of each procedure.

**Results:**

Before or after matching, CN led to better OS and lower CSM in Kaplan-Meier analysis. In matched cohort, decreased CSM after CN compared to without CN were consistently found in most subgroups stratified by age, T stage, and patients with ≤2 site-specific metastases. However, patients with ≥ 3 site-specific metastases, or patients with ≥cT3 stage combined with ≥ 2 site-specific metastases were not benefit from the cytoreductive nephrectomy.

**Conclusions:**

The potential benefit of CN disappeared in patients with ≥ 3 site-specific metastases, or patients with ≥cT3 combined with ≥ 2 site-specific metastases.

## Introduction

Metastatic renal cell carcinoma (RCC) accounts for one-third of the total RCC[1]. Systemic treatments, including immunotherapy and targeted therapies, are commonly used for these patients. Cytoreductive nephrectomy (CN) is most often performed with the goal of achieving palliation from symptoms or reduction the primary gross tumor before or after systemic treatment as part of integrated management strategy[2]. Some published data have reported the benefit of CN in immunotherapy era with improved overall survival (13.6 vs 7.8 mo) [3–5], as well as in the setting of patients with targeted therapy [6–9]. However, these studies also point toward the importance of selecting patients who should undergo CN, as there are certain subgroups of patients with mRCC who may not benefit from CN. Indeed, there has been significant uncertainty in the selection of candidates for CN, and the impact of CN on survival might be largely influenced by primary tumor and metastases characteristics[6]. Currently CN is recommended in mRCC patients with a good performance status, large primary tumors and low metastatic volume[2], although recently the CARMENA trial showed that sunitinib alone was not inferior to nephrectomy followed by sunitinib in patients with mRCC who were classified as having intermediate-risk or poor-risk disease[10].

Patients with different metastatic sites might represent different subgroups of patients with different tumor biologic patterns and prognosis and subsequently, distinct therapeutic approaches. Previous study has reported that the most frequent site of metastases was lung (54%)[11]. Some cases could undergo a change of the metastatic pattern and involve other distant organs. Previous study had pointed out that liver metastases might be a predictor for poor prognosis in metastatic renal carcinoma patients[12]. Multiple sites of metastases were associated with worse survival in patients with malignant melanoma of the skin and pancreatic adenocarcinoma[13,14]. For other metastatic cancers, radical cystectomy and prostatectomy only did bring benefits for survival in metastatic bladder cancer with single metastatic sites but not with multiple metastatic sites [15] and in metastatic prostate cancer patients only with oligometastatic sites [16]. Nevertheless, due to the limited number of patients and differences in treatment strategies, little data existed for investigating the influence of site-specific metastases (bone metastases, liver, brain or lung metastases) on CN or not for mRCC patients. This is important, because the response to both CN and systemic therapy can be variable, with some patients undergoing rapid progression of their disease despite multimodal therapy.

## Methods and patients

### Study population and data collection

A retrospective cohort was identified by use of the Surveillance, Epidemiology, and End Results (SEER) database, which is sponsored by the National Cancer Institute (www.seer.cancer.gov). Because this study is based on a publicly available database, it was exempted from ethical approval. We retrieved data using the SEER*Stat software Version 8.3.4. We restricted our search to SEER database between 2010 to 2014.Within the SEER database, we identified patients diagnosed with primary kidney cancer (ICD-O-3 site codes C64.9) with metastatic disease at diagnosis (ie, SEER field “CS Mets at DX”). Patients with cytoreductive nephrectomy codes (codes 40,50,70) or not surgery (code 0) were selected. Patients with only complete information on age, T stage, size, site of metastases, histology, grade, survival months and vital status were included in the study. Additionally, patients with less than 3 months’ survival time after diagnosis were excluded to reduce the bias survival as much as possible due to patients with significant comorbidity or very bad performance status. Site-specific metastases included liver, lung, bone, and brain. TNM stage was manually recoded according to SEER variables (CS tumor size, lymph nodes, extension 2004+) and the AJCC 8th edition tumor-nodes-metastasis (TNM) classification.

### Statistical analysis

Due to potential differences in patients undergoing cytoreductive nephrectomy or not, the propensity score-matching was used to balance the potential probability of being assigned. The age at diagnosis, clinical T stage (T1-2 or T3-4), pathological grade, and number of site-specific metastases were used to calculate the propensity scores. Mean propensity-score for CN group was 0.136, and 0.221 for no CN group (p<0.001). 1: 1 pair matching without replacement was implemented by nearest neighbor matching method with caliber of 0.02. After matching, 168 pairs of patients were included after applying propensity scores, and there was no significant difference in mean propensity-score between two group, and all standardized differences were well below 10%.

Outcomes of interest included overall survival (OS) and cancer-specific mortality (CSM), as well as factors independently associated with CSM. Kaplan-Meier methods were used to determine OS and CSM. Competing risks regression analysis, according to the model of Fine and Gray[17], was used to calculate the cumulative incidence of RCC-specific death using death from non-RCC as the competing variable. Stepwise multivariable competing risks regression analysis was used to identify factors independently associated with CSM using backward elimination of variables based on the Wald test. Demographic, clinical and pathologic data were compared using independent t-tests for continuous variables and Pearson’s chi-square tests for categorical variables.

Secondary subgroup analyses in the PSM matched cohort were performed using univariate Cox proportional hazard model estimated the HRs of CN versus no surgery and a table was showed to better present each prognostic factor’s effect on CSM, which stratified to patients of different age (≤ 65, > 65 years), T stage (T1-2, T3-4), number. Site-specific metastases (≤1, 2, 3–4), and ≥cT3 combined with multiple site-specific metastases(no, yes).

Statistical analysis was performed with the Statistical Package for the Social Sciences Software (SPSS version 23, IBM Corp, Armonk, NY). A two-sided p value of less than 0.05 was considered statistically significant.

## Results

A total of 1113 eligible cases were identified: No surgery (NS) (n = 495), and CN (n = 618). A total of 702 patients (63.1%) died of mRCC and 54 patients (4.8%) died of other causes. Of these cancer-related deaths, 376 of 495 (76.9%) occurred in the NS group and 326 of 618 (52.7%) in the CN group. Patient characteristics are listed in **Table 1**. CN patients seem to be younger, less likely to have positive regional lymph node, and less sites metastases, but more likely to have higher T grade (each p<0.0001). Specially, in all patients 662 (59.5%) patients were diagnosed with lung metastases, 421 (37.8%) patients were diagnosed with bone metastases, 179 (16.1%) patients were diagnosed with liver metastases, and 122 (10.9%) patients were diagnosed with brain metastases. A total of 711 (63.9%) patients have a less than one specific metastases organ while 248 patients (22.2%) patients have two specific organ metastases and 79 (7.1%) have multiple organ metastases. After propensity-score matching, preoperative characteristics were well balanced (**Table 1**).

**Table 1:** Patient baseline demographics and pathological characteristics. Full sample and propensity score matched cohorts.

Characteristic		Full Sample			Matched Cohort		
		No surgery	Cytoreductive nephrectomy	p value	No surgery	Cytoreductive nephrectomy	Std. Diff(%)
		**N = 495**	**N = 618**	**-**	**N = 168**	**N = 168**	
**Tumor size, cm(SD)**	**-**	**8.5(4.2)**	**9.9(4.8)**	**<0.001**	**8.5(3.9)**	**8.6(4.1)**	**0.1**
**Median age, yr (SD)**	**-**	**65.5(12.3)**	**59.4(10.9)**	**<0.001**	**60.5(9.1)**	**61.2(9.8)**	**0.7**
**Age at diagnosis (years)**	**≤65**	**256(51.7)**	**435(70.4)**	**<0.001**	**91(54.2)**	**88(52.4)**	**-1.8**
	**>65**	**239(48.3)**	**183(29.6)**		**77(45.8)**	**80(47.6)**	**1.8**
**Sex, no. (%)**	**Female**	**142(28.7)**	**163(26.4)**	**0.39**	**54(32.2)**	**56(33.3)**	**1.1**
	**Male**	**353(71.3)**	**455(73.6)**		**114(67.8)**	**112(66.7)**	**-1.1**
**Race, no. (%)**	**White**	**377(76.2)**	**487(78.8)**	**0.044**	**126(75.0)**	**120(71.4)**	**-3.6**
	**African American**	**60(12.1)**	**48(7.8)**		**22(13.1)**	**24(14.3)**	**1.2**
	**Other**	**58(11.7)**	**83(13.4)**		**20(11.9)**	**24(14,3)**	**2.4**
**Marital status, no. (%)**	**Married**	**269(54.3)**	**408(66.0)**	**<0.001**	**92(54.7)**	**86(51.2)**	**-3.6**
	**Single/widowed/divorced**	**213(43)**	**139(31.2)**		**69(41.0)**	**74(44.0)**	**3.0**
	**Unknown**	**13(2.6)**	**17(2.8)**		**7(4.3)**	**8(4.8)**	**0.6**
**Laterality, no. (%)**	**Left**	**258(52.1)**	**328(53.1)**	**0.752**	**99(58.9)**	**99(58.9)**	**0.0**
	**Right**	**237(47.9)**	**290(46.9)**		**69(41.1)**	**69(41.1)**	**0.0**
**Tumor grade, no. (%)**	**Well+ moderate**	**34(6.9)**	**109(17.1)**	**<0.001**	**33(19.6)**	**41(24.4)**	**4.6**
	**Poor +undifferentiated**	**61(12.3)**	**444(71.8)**		**59(35.1)**	**62(36.9)**	**1.9**
	**Unknown**	**400(80.8)**	**65(10.5)**		**76(45.3)**	**65(38.7)**	**-6.5**
** Histology type, no.(%)**	**Clear-cell**	**200(40.4)**	**399(64.6)**	**<0.001**	**78(46.4)**	**72(42.9)**	**-3.6**
	**Non-Clear-cell**	**295(59.6)**	**219(35.4)**		**90(53.6)**	**96(57.1)**	**3.6**
**Clinical T stage, no. (%)**	**T1**	**139(28.1)**	**71(11.5)**	**<0.001**	**45(26.7)**	**43(25.6)**	**-1.2**
	**T2**	**138(27.9)**	**84(13.6)**		**51(30.3)**	**46(27.4)**	**-3.0**
	**T3**	**136(27.5)**	**391(63.3)**		**50(29.7)**	**54(32.1)**	**2.4**
	**T4**	**82(16.6)**	**72(11.7)**		**22(13.3)**	**25(14.9)**	**1.8**
**Clinical N stage, no. (%)**	**N0**	**279(56.4)**	**426(68.9)**	**<0.001**	**88(52.4)**	**88(52.4)**	**0.0**
	**N1**	**174(35.2)**	**166(26.9)**		**53(31.6)**	**58(34.5)**	**3.0**
	**NX**	**42(8.5)**	**26(4.2)**		**27(16.0)**	**22(13.1)**	**-3.0**
**Metastases site, no. (%)**	**Lung**	**304(61.4)**	**358(57.9)**	**0.239**	**107(63.7)**	**112(66.7)**	**3.0**
	**Liver**	**100(20.2)**	**79(12.8)**	**0.001**	**22(13.1)**	**22(13.1)**	**0.0**
	**Bone**	**221(44.6)**	**200(32.4)**	**<0.001**	**65(38.7)**	**63(37.5)**	**-1.2**
	**Brain**	**72(14.5)**	**50(8.1)**	**0.001**	**27(16.1)**	**27(16.1)**	**0.0**
**Single metastases site, no. (%)**	**Lung**	**135(50.9)**	**236(61.9)**	**0.024**	**41(24.4)**	**44(26.2)**	**1.8**
	**Liver**	**20(7.5)**	**25(6.6)**		**15(8.9)**	**17(10.1)**	**1.2**
	**Bone**	**92(4.7)**	**107(28.1)**		**25(14.9)**	**27(16.1)**	**1.2**
	**Brain**	**18(6.8)**	**13(3.4)**		**14(8.3)**	**13(7.8)**	**-0.6**
**Number of site-specific metastases, no. (%)**	**1**	**262(52.9)**	**449(72.7)**	**<0.001**	**85(50.6)**	**84(50)**	**-0.6**
	**2**	**141(28.5)**	**107(17.3)**		**53(31.5)**	**55(32.3)**	**1.2**
	**3–4**	**49(9.9)**	**30(4.8)**		**30(17.9)**	**29(17.7)**	**-0.6**

**Fig 1:**
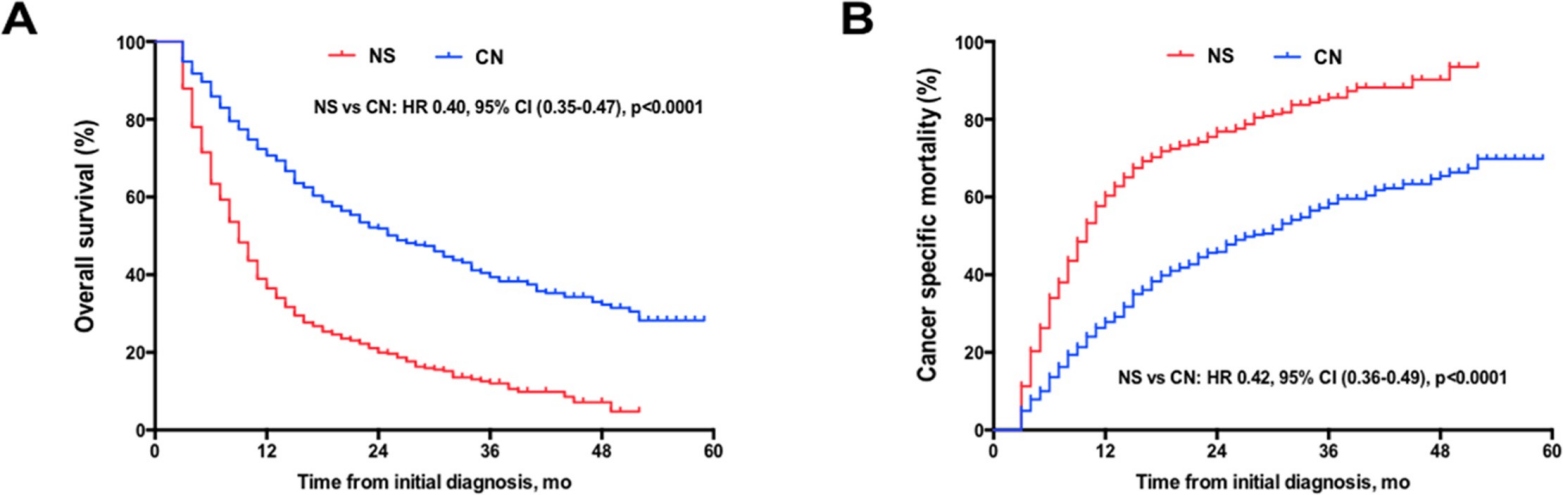
(A) Overall survival and (B) cumulative incidence of renal cell carcinoma (RCC)-specific mortality in patients with metastatic RCC at diagnosis based on treatment received in full samples. NS = no surgery therapy; CN = cytoreductive nephrectomy.

Before matching, the median overall survival was 26 months (95%CI [22.1–29.7]) in the CN group and 9 months (95% CI 8.1–9.8) in the NS group. The relative risk of death was 60% lower in the CN group than in the NS group (HR, 0.40; 95% CI [0.35 to 0.47]; p<0.0001). The 1-yr OS was significantly higher in patients undergoing cytoreductive nephrectomy compared with NS patients (70.7% vs. 43.6%, p<0.001) (**Fig 1A**). Correspondingly, the medians cancer-specific survival was much longer in CN group compared to NS group (28 months vs. 10 months, p<0.001). The CSM were lower in CN patients (HR: 0.42, 95% CI: 0.36–0.49, p<0.0001) than in NS patients. The 1-yr CSM was lower in patients undergoing CN than in patients without CN (27.9% vs. 60.3%) (**Fig 1B**). In addition, in 51 patients dying of non-mRCC causes (35 in NS and 19 in CN group), no significant differences in survival were noted among groups. Therefore we focused on the CSM in the following analysis. On multivariate analysis, CN was an independent favor predictor of CSM. Additionally, the multivariate analysis showed that age at diagnosis, T stage, histology subtype, and number of multiple-sites specific metastases were associated with outcomes (**Table 2**).

**Table 2:** Stepwise multivariable competing risks regression analysis of cancer specific mortality in patients with metastatic RCC at diagnosis.

Characteristic	Full Sample			Matched Cohort		
Characteristic	Adjusted HR	95% CI	p value	Adjusted HR	95% CI	p value
**Type of treatment**						
** No surgery**	**Ref**			**Ref**		
** Cytoreductive nephrectomy**	**0.42**	**0.36–0.49**	**<0.001**	**0.70**	**0.51–0.89**	**<0.001**
**Age, yr**						
** ≤65**	**Ref**			**Ref**		
** >65**	**1.18**	**1.01–1.38**	**0.045**	**1.31**	**1.26–1.36**	**0.021**
**Histology type**						
** Clear-cell**	**Ref**			**Ref**		
** Non-Clear-cell**	**1.13**	**1.03–1.22**	**0.005**	**1.09**	**0.99–1.19**	**0.052**
**T stage**						
** T1-T2**	**Ref**			**Ref**		
** T3-T4**	**1.45**	**1.24–1.70**	**<0.001**	**1.64**	**1.53–1.75**	**<0.001**
**Number of site-specific metastases**						
** ≤1**	**Ref**			**Ref**		
** 2**	**1.73**	**1.31–2.27**	**<0.001**	**1.6**	**1.42–1.78**	**<0.001**
** 3–4**	**2.7**	**2.09–3.50**	**<0.001**	**3.1**	**2.20–4.0**	**<0.001**

CI = confidence interval, HR = Hazard Ratio

**Fig 2:**
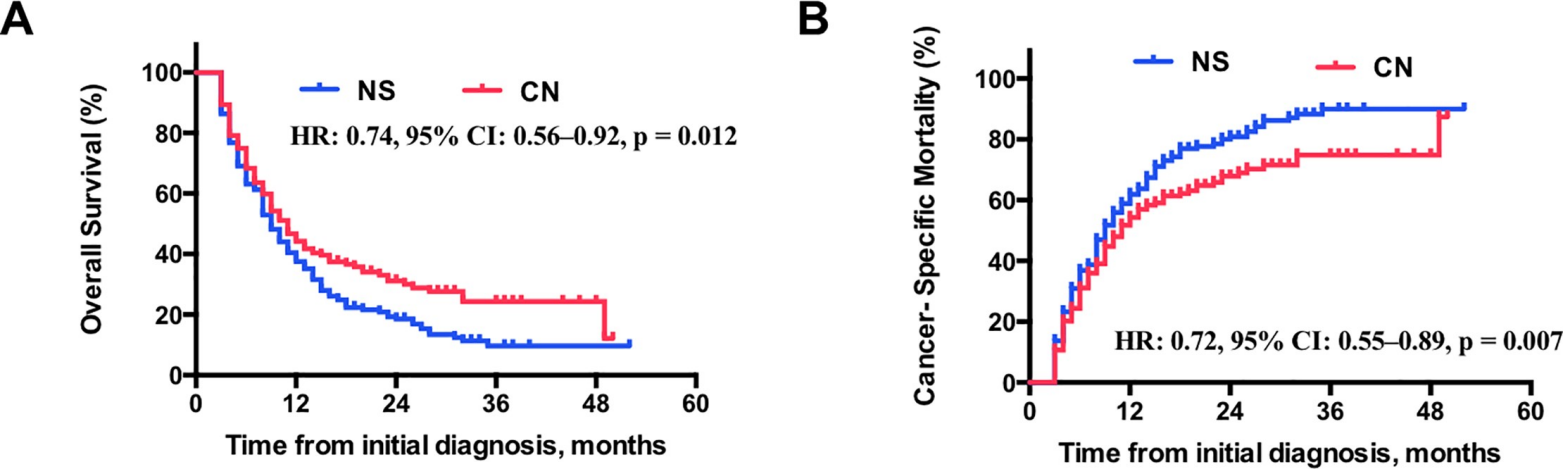
(A) Overall survival and (B) cumulative incidence of renal cell carcinoma (RCC)-specific mortality in patients with metastatic RCC at diagnosis based on treatment received in matched cohort. NS = no surgery therapy; CN = cytoreductive nephrectomy.

Within the propensity-score matched cohort, CN was still associated with significant better outcomes in OS (HR: 0.74, 95% CI: 0.56–0.92, p = 0.012), and lower CSM (HR:0.72, 95% CI: 0.55–0.89, p = 0.007) compared with NS patients (**Fig 2**). The multivariate analysis also validated that age at diagnosis, T stage and number of multiple-sites specific metastases were associated with risk of CSM (**Table 2**). The secondary analysis based on the matched cohort was further studied when the patients were stratified by these risk factors. Decreased CSM after CN were consistently found in most subgroups stratified by age, T stage, and patients with ≤2 site-specific metastases. However, patients with ≥ 3 site-specific metastases, or patients with ≥cT3 stage combined with multiple site-specific metastases were not benefit from the cytoreductive nephrectomy (**Table 3**). However, whether the cytoreductive nephrectomy can relieve symptoms and improve quality of life is unknown according to the SEER database.

**Table 3:** Subset analyses of CSM in matched cohort between cytoreductive nephrectomy versus no surgery using univariate Cox proportional hazard model.

Variables		HR	95% CI	p value
Age, yr				
	≤65	0.55	0.38–0.72	<0.001
	>65	0.89	0.84–0.94	0.026
T stage				
	T1-T2	0.46	0.39–0.51	<0.001
	T3-T4	0.88	0.79–0.97	0.037
Number. Site-specific metastases				
	≤1	0.34	0.31–0.37	<0.001
	2	0.68	0.46–0.84	0.0002
	3–4	0.89	0.47–1.31	0.566
≥cT3 + multiple site-specific metastases				
	no	0.59	0.47–0.71	p<0.001
	yes	1.06	0.79–1.33	p = 0.479

## Discussion

Clear evidence of the benefit provided by CN in the metastatic renal carcinoma patients is lacking, but nonrandomized evidence suggests a possible survival advantage for this approach, which was reviewed in a meta-analysis[18]. Indeed, strict criteria for selecting candidates originated from most studies, namely, which included only those patients with a good performance status or excluded patients with symptomatic or untreated brain metastases or those with exclusive bone metastases or multiple metastases at one single organ. These stringent inclusion and exclusion criteria mean that upcoming results from these trials will not provide data that are generalizable to real world practice. Several studies have focused on the survival outcomes in patients with metastases in the bone[9,19], liver[19], lung[20], and brain[21,22] and higher tumor grades [23], and found them as negative predictors of OS. However, no study paid attention to the important role of the number of metastatic sites on the prognosis of patients with mRCC.

Careful patient selection for CN is critical because those with poor survival outcome or who are likely to progress rapidly may receive minimal benefit. You et al[24] identified 4 preoperative risk factors (Karnofsky performance status, hemoglobin, neutrophils, and clinical N stage) for the selection of patients undergoing CN and only those with 0 to 1 risk factors can derive benefit compared with those who received systemic therapy alone. In another study, Heng et al[6] demonstrated that patients with estimated survival times <12 months or four or more IMDC prognostic factors may not benefit from CN. Culp et al.[25] identified seven pre-operative variables that permitted them to distinguish patients who were unlikely to benefit from CN: serum albumin and lactate dehydrogenase levels, clinical stage T3 or T4, symptoms caused by metastatic spread, liver metastasis and radiographic evidence of retroperitoneal or supradiaphragmatic adenopathy. Surgical patients who had ≥4 risk factors did not appear to benefit from CN. From the same database, Margulis et al.[26] developed a pre-operative nomogram, including serum albumin and lactate dehydrogenase levels, to aid identification of patients with mRCC who would or would not benefit from CN. Therefore, it is very important to select more appropriate patients to receive CN in more appropriate time.

Several of our findings are noteworthy. In our study, number of metastatic sites seemed to be related with survival. We are the first to report the efficacy and benefit of the CN in patients with multiple site-specific metastases. Consistent to metastatic bladder cancer, radical cystectomy was an independent predictor for better overall survival and cancer-specific survival, while in patients with multiple metastatic sites, radical cystectomy did not bring benefits[15]. In metastatic prostate cancer, patients with oligometastatic sites (low number of metastases) could still benefit from radical prostatectomy [16]. The effect of metastatic sites on patients’ prognosis also had been discussed in other several different cancers, such as breast cancer[27] and pancreatic adenocarcinoma[14].

Importantly, we also identified age>65 years, ≥cT3, multiple site-specific metastases were independent pre-operative risk factors of mortality. Using the propensity-score matched cohort, we compared CSM between the CN and non-CN groups according to the number of pre-operative risk factors. We identified that patients with ≥3 site-specific metastases, or patients with both ≥ cT3 and multiple site-specific metastases were not benefit from the cytoreductive nephrectomy. A previous study also concluded that rigorous patient selection is essential, as elderly patients, patients with significant comorbidities, or patients with tumors >14 cm have higher risk of perioperative mortality, which may outweigh the survival benefit [28]. Although recently the CARMENA trial showed that sunitinib alone was not inferior to nephrectomy followed by sunitinib in patients with mRCC who were classified as having intermediate-risk or poor-risk disease[10], we still should be aware that it was a noninferiority trial, the results may underestimate the benefit of nephrectomy.

The current study is not without limitations. First, the analyses are retrospective in nature; this comes with an unavoidable selection bias that is prevalent in all non-prospective, nonrandomized studies. Also, the treating physician's perception of the patient's prognosis rather than the actual severity of their disease may have limited what treatment patients were offered. Second, the results of this analysis have to be interpreted with caution of the lack of information about the co-morbidities, performance status, actual metastatic tumor volume, and the IMDC and MSKCC prognostic variables in evaluated patients. In the absence of these data points, the selection bias in choosing patients for surgical procedures is not known. Despite these limitations, information on important patient-related and tumor-related factors that play a vital role in the decision making was available and analyzed in this study.

In conclusions, our findings demonstrate that although perioperative mortality is significant, CN may provide an OS benefit in mRCC patients. Patients with ≥3 site-specific metastases or those with ≥cT3 stage combined with multiple site-specific metastases may not receive a substantial benefit. Stringent patient selection remains vital as we await results from the randomized controlled trials.

## Abbreviations

CN: cytoreductive nephrectomy
mRCC: metastatic renal carcinoma
CSM: cancer-specific mortality
OS: overall survival
